# Translation of Cultures and Texts: Envisioning a Culturally Responsive Translational Practice in Qualitative Research

**DOI:** 10.1177/10497323251316757

**Published:** 2025-04-02

**Authors:** Pengfei Zhao, Pei-Jung Li, Wen Qi

**Affiliations:** 1 Department of Integrated Studies in Education, Faculty of Education, 5620 McGill University, Montreal, QC, Canada; 2 Department of Applied Psychology in Education and Research Methodology, School of Education, 1772 Indiana University Bloomington, Bloomington, IN, USA; 3 Office of the Vice Provost for Faculty and Academic Affairs, 1772 Indiana University Bloomington, Bloomington, IN, USA

**Keywords:** culturally responsive research, translation, cross-cultural research, cross-lingual research, translators

## Abstract

In this methodological paper, we raise the question of what a culturally responsive translational practice might look like in qualitative research. Through examining the literature on translation in culturally responsive theories and qualitative research methodology, we distinguish two approaches in addressing the issue of translation: translation as texts and translation as cultures. To enact a culturally responsive translational practice, qualitative researchers should maintain an intimately linked dual-focus in their work, attending to both the practical aspects of translation that directly lead to the production of the final translated texts, as well as translation’s multi-layered cultural and political effects. This proposal is further unpacked on three levels: (1) On the level of social and cultural processes and structure, we examine the routes and gatekeepers of translation in the context of knowledge production and mobilization; (2) on the level of intersubjective relationality, we explore the significance of visibilizing translation and translators; and (3) on the level of human–text interaction, we consider how interpretive approaches, untranslatability, and styles of translation may shape researchers’ translation practice. While drawing insights from culturally responsive theories, we also substantiate our argument using critical translational studies and examples from our empirical research projects. Taken together, this paper outlines some important considerations qualitative researchers should take into account as they envision a culturally responsive translational practice in qualitative research and calls for researchers to engage in this work with multilingual awareness, reflexivity, and criticality.

## Introduction

Indeed, the process of translation (Freeman, 2009; Temple, 1997) is epistemologically layered. Meaning is created and recreated at multiple ethnographic moments and by multiple people throughout the process. The process of translation thus raises questions of authorship, authority, power, status, relationship, and voice, all immensely relevant and powerful concerns in culturally responsive practice. (Chouinard & Cram, 2019, p. 111)

Culturally responsive theories have underscored our understanding of culture(s) in qualitative research, particularly when researchers work with marginalized and minoritized research participants in non-Western, cross-cultural, and transnational contexts (Berryman et al., 2013; Chouinard & Cram, 2019; Jordan & Hall, 2023). In many cases, the discussion of cultures is inseparable from the use of different languages and thus the enactment of translation. As Chouinard and Cram (2019) point out in the quote above, culturally responsive theorists have raised thought-provoking questions on the role of translation when it comes to the interrogation of power relationships, the re/presentation of voices, and the researcher–participant relationship across different cultures and mediated by various languages (Chouinard & Cram, 2019). Much of the discussion resonates well with contemporary translation studies, which widely explores how various translational practices have perpetuated language hierarchy and cultural hegemony in an era of globalization (Apter, 2014; Bhabha, 2012; Pym, 2014; Venuti, 2008).

Within the field of qualitative inquiry, researchers are still most concerned with the practical-technical aspect of translation and have yet to take up the insights from culturally responsive theories (e.g., Abfalter et al., 2021; Cortazzi et al., 2011; Kalocsányiová & Shatnawi, 2021; Qun & Carey, 2024; Sutrisno et al., 2014). Indeed, these two sets of literature—culturally responsive theories and qualitative methodology—have, for the most part, addressed translation separately, with limited convergence or dialogue. Given the foundational role that translation plays in cross-language and cross-cultural research (Temple & Young, 2004), we argue that cultivating a dialogue between culturally responsive theories and qualitative methodology could be beneficial in expanding our current research practice regarding translation. This is especially true for qualitative health researchers, considering the growing number of linguistically minoritized individuals served by medical professionals. For instance, in the United States alone, around 8.5% of the population, or around 26 million people, are classified as having limited English proficiency (LEP), meaning they either do not speak English or do not speak it well (U.S. Census Bureau, 2023). A key objective for qualitative health researchers is to explore ways to improve medical services for such populations, ensuring that care is delivered with cultural awareness and respect. This requires health researchers not only to promote clear and effective communication between health care providers and patients (The National Council on Interpreting in Health Care, 2004) but also to enhance patient and public involvement and engagement in research—an essential factor in achieving equitable and dignified care (Rolfe et al., 2018).

In this paper, we raise the following question: *What might a culturally responsive translational practice look like in qualitative research?* Under this overarching question, we explore informative methodological concepts and their relationships, as well as the underlying ethical and epistemological tenets that can shed light on the still largely invisible practice of translation in qualitative inquiry. In particular, using examples from our own empirical studies, we examine the moments when translation becomes a point of contestation/contention in interpreting and negotiating meaning, tease out the interlaced relationship between cultures and languages, and finally, explore reflexive approaches that qualitative researchers can engage in to cultivate multilingual awareness and criticality.

As we outline our methodological proposal, we offer the following secondary questions to address our central concern regarding culturally responsive translational practice on three levels:

*Social and cultural processes and structure*: When qualitative researchers are asked to be culturally responsive in cross-cultural research that involves translation, which culture(s) should they reference and account for, and what institutionalized gatekeepers and structural barriers should qualitative researchers attend to while engaging in culturally responsive translational practice?
*Interpersonal interaction/relationality*: Since translators are often considered cultural brokers in cross-cultural communication (Jijon, 2019), how might culturally responsive theories inform us about the role of translators in qualitative research beyond brokerage, and what does recognizing translators as more-than-brokers imply about the relationship among researchers, translators, and participants? Additionally, critical qualitative researchers have been advocating for making both the translation and translator in qualitative research visible (Burkhard & Park, 2024; Temple & Young, 2004; Wong & Poon, 2010). How might increasing the visibility of translation and translators enhance culturally responsive research practice?
*Researchers/translators–texts interaction:* What specific translational practices can qualitative researchers engage in in their daily research, and what are the cultural and political implications for these practices?

These questions invite us to first compare and juxtapose the distinctive approaches that culturally responsive theories and qualitative methodological literature have been engaging in to address the question of translation and, subsequently, build a three-dimensional dialogue between the two sets of literature.

Before we dive into the discussion, we, the three authors, would also like to offer a brief discussion of our positionality as we grapple with the questions raised above. All three of us self-identify as transnational and multilingual qualitative methodologists/researchers. We share a migratory background of moving from East Asia to North America in pursuit of graduate degrees. All three of us speak multiple languages of varying proficiency levels, including, but not limited to, Mandarin Chinese and English. Some of our empirical research involves working with multicultural and multilingual participants such as international students and new immigrant families in transnational contexts, and some of it involves engaging with participants who primarily speak Mandarin Chinese. Up until now, the majority of our research findings have been published in English, but we do participate in knowledge mobilization activities in Mandarin Chinese and translanguaging/multilingual contexts. Throughout our cross-cultural and cross-lingual work, we have identified moments where our translational practices could have been better supported by culturally responsive theories, some of which we share in the following sections. More importantly, dwelling in a liminal space between different languages and cultures—where the translation of both texts and cultures is constantly negotiated—is not only essential to our research but also an inescapable aspect of our living condition. All of this lived experience and professional engagement has enriched our understanding of translation and motivated us to delve deeper into its methodological implications.

## Translation at the Intersection of Culturally Responsive Theories and Qualitative Methodological Literature

In a comprehensive review of the various versions of cultural translation, Anthony Pym distinguishes “the translation of texts” from “the translation of cultures” (Pym, 2014, p. 139). Pym points out that “the translation of texts” is concerned with the relationship between a start/original text (i.e., the text to be translated) and its corresponding target/translated text with a purposive orientation toward the products of translational practice, whereas “the translation of cultures” can be understood as “a process in which there is no start text and usually no fixed target text. The focus is on cultural processes rather than products” (2014, p. 139). If we take a closer look at how culturally responsive theories and qualitative methodological literature address the issue of translation, we may find that the former often grapples with “the translation of cultures” and the latter primarily focuses on “the translation of texts.”

### Translation as “the Translation of Texts”

Qualitative research has a long tradition of examining the role of language in relation to the representation of the external world, one’s lived experience, and intersubjectivity (e.g., Carspecken, 2003; Denzin, 2002; Polkinghorne, 2005). Previous researchers have explored this issue from the philosophic perspectives of phenomenology and hermeneutics (Freeman & Vagle, 2013), critical theories (Carspecken, 2003), post-structuralism and post-modernism (MacLure, 2013), as well as language’s intricate relationship with research ethics (Smith, 2016; Spivak, 2023). Generally speaking, the discussions of language in philosophic and theoretical discourses presume a universalized significance in addressing the nature of meaning and reference, intentionality, and the relationship among language, its users, and the world. Language in research practice, however, often requires qualitative researchers to delve into a much more complex linguistic landscape, including but not limited to the use of more than one language. Translation in the overall research methodology literature thus is constructed as a practical, “niche” issue, first and primarily a set of questions regarding specific, cross-cultural research scenarios.

The practical challenges that translation presents for some qualitative researchers necessitate addressing it as what Pym and others refer to as “the translation of texts.” Qualitative methodologists have meticulously studied a wide range of issues including meaning-making in translational practice in qualitative research (e.g., Larkin et al., 2007; Temple & Young, 2004; Wong & Poon, 2010); language choices and the quality of data (e.g., Cortazzi et al., 2011); the validity and fidelity of translation (e.g., Chen & Boore, 2010; Halai, 2007; Kapborga & Berterö, 2002; Sutrisno et al., 2014; Twinn, 1997; Zhao et al., 2024); the role of translators in relation to researchers and research participants (e.g., Gawlewicz, 2016; Kalocsányiová & Shatnawi, 2021; Kim, 2012; Qun & Carey, 2024); and the timing and strategies of translation (e.g., Abfalter et al., 2021; Gawlewicz, 2016; Inhetveen, 2012; Turhan & Bernard, 2021). Indeed, many of the discussions oriented themselves toward the pursuit of equivalence as defined by the identification of the meanings between the original and the translated texts (Zhao et al., 2024). With a few exceptions (e.g., Burkhard & Park, 2024; Temple & Young, 2004), researchers adopting this approach have devoted relatively limited space to addressing macro-level structural issues, such as language hierarchies, language ideologies, and cultural/epistemological hegemony.

Qualitative health researchers have a particularly keen awareness of the complex multilingual and/or cross-lingual contexts which they often need to navigate. These contexts include but are not limited to field sites such as health care institutions, clinical settings, communities of linguistically minoritized, immigrant families, and countries and regions where the primary language used is not English (e.g., Björk Brämberg & Dahlberg, 2013; Espinosa et al., 2022; Sanderson et al., 2013; Tsai et al., 2004). Additionally, researchers find themselves addressing translational issues when collaborating with international research teams (Espinosa et al., 2022; Ho et al., 2019; Santos et al., 2015; Varshney et al., 2016), transferring research instruments (e.g., questionnaires) from one cultural context to another (Jagosh & Boudreau, 2009; Lor & Gao, 2020), and reporting the translation process (Yunus et al., 2022). Many discussions give actionable advice to facilitate qualitative researchers’ methodological decision-making. For example, Santos et al. (2015) discuss the effect of the timing of the translation in international research collaboration and how it interacts with the products of translation (e.g., instruments, interview questions, data, and analytical concepts) and translator competence. They recommended that translation in qualitative health research should occur in the early phases of a study rather than the later phase such as at the time of the dissemination of findings. They also suggest keeping an audit trail of translation decisions to ensure interpretation transparency (Santos et al., 2015; van Nes et al., 2010). In another example, based on their work with Swedish home care receivers, Björk Brämberg and Dahlberg (2013) offer suggestions about working with interpreters in interview-based studies. They advise that an interpreter should use not only the verbatim translation but also the general contexts to help researchers to more accurately understand and present the overall meanings. This would require the interpreters to “understand the context of the questions and make a validity claim of the words in their own culture” (p. 243). Although many qualitative health researchers recognized the significance of culture in translation, culture is often framed in relation to the final product of translation, as a factor that impacts the quality of translation (Espinosa et al., 2022; Ho et al., 2019; Lor & Gao, 2020), or a challenge posed to both translators and researchers (Choi et al., 2012; Esposito, 2001; Squires, 2008).

### Translation as “the Translation of Cultures”

Culturally responsive researchers have been responding to the practical orientation of the methodological literature on translation, most noticeably from the perspectives of cultural and political critique. For instance, Chouinard and Cram (2019) note that much of the methodological literature on translation is too technical to facilitate investigations into translation’s cultural and political effects and implications. They contend that not all of the methodological challenges regarding translation have been sufficiently addressed and that most of the issues are undertheorized. Performing empirical research in both Western and non-Western contexts, culturally responsive researchers are keen to point out the limitations of the “translation as texts” approach (Janusch, 2011), especially when these limitations are manifested in constructing research instruments (Marcos et al., 1973) and communicating with participants nonverbally or in dialects and colloquiums (Karidakis, 2021).

The works by culturally responsive theorists serve as a welcoming call to qualitative researchers, reminding them that translation is not merely the search for equivalence between the original and the target texts but also “the translation of cultures” in the context of generating and disseminating knowledge. Translation does not take place in one or two designated research stages/procedures, nor does it happen in a way that can be segregated from other research activities. The use of languages, such as who is speaking in what language(s), who can or cannot decide on the translation of specific utterances, and for what purposes the translated words are communicated, is inherently political in the liminal space of cultural encounters.

Indeed, “cultural encounters” as a sensitizing concept or an illustrative metaphor has taken center stage in culturally responsive theorists’ theorization. The term “culture,” however, has undeniably been one of the most important, yet also contentious and meaning-loaded, keywords in contemporary social research (Bourdieu, 1986; Freire, 2018; Geertz, 1973; Giddens, 1979; Gramsci, 2005; Ortner, 1984; Williams, 2014). It is thus beneficial for us to briefly revisit culturally responsive theorists’ approach to “culture” in order to unpack the meaning of the translation of cultures.

Generally speaking, culturally responsive theorists (Berryman et al., 2013; Chouinard & Cram, 2019; Jordan & Hall, 2023) align themselves with critical theories’ approaches to conceptualizing culture (Freire, 2018; Gramsci, 2005; Williams, 2014), and also highlight the importance of the non-Western, decolonizing, and indigenous perspectives on cultures (e.g., Smith, 2016; Spivak, 2023). For instance, the works by Raymond Williams (2014) and Antonio Gramsci (2005) make strong arguments about the limitations of an interpretivist approach to the study of cultures (e.g., Geertz, 1973), namely, that it is not enough for researchers to merely decipher the symbolic significance/meaning of social actions from the perspective of the social members who belong to and practice that very culture. What matters more is to interrogate how culture is valorized by the dominant social group in order to manipulate and control the subordinate groups. The questions of domination and suppression, and power and empowerment, are thus at the very core of a critical analysis of culture.

Additionally, culturally responsive theorists argue that culture contributes to the formation of social groups’ collective identities and the demarcation of the self-other relationship. The other is thus self-constitutive; that is, we make sense of who we are as individuals and groups through understanding who we are not. For example, drawing on Kaupapa Māori Theory, Berryman et al. (2013) adopt an informed and holistic approach to culture:

Culture is what holds a community together, giving a common framework of meaning. […] Culture is preserved in language, symbols and customs and celebrated in art, music, drama, literature, religion and social gatherings. It constitutes the collective memory of the people and the collective heritage which will be handed down to future generations. (Quest Rapuara, as cited by Berryman et al., 2013, p. 4)

While critical theorists and indigenous scholars often develop their approaches to culture through an overarching critique of the modern Western society, culturally responsive theorists situate their examination of the encounter of cultures in the context of doing social and applied social research (Berryman et al., 2013; Chouinard & Cram, 2019; Jordan & Hall, 2023). Specifically, in the realm of knowledge production, the self–other relationship manifests in the relationship between researchers and their research participants. Such a relationship can be highly complex and asymmetrical, particularly under the dominant objectivist and post/positivist framework. In these contexts, research participants are often subject to the Western gaze (Mohanty, 2003; Smith, 2016). This can also lead to an othering process in which minoritized communities are continuously being exploited, extracted, and marginalized (Denzin & Giardina, 2019; Sandoval, 2013; Smith, 2016; Spivak, 2023; Swadener & Mutua, 2008).

Culturally responsive theorists demonstrate to us that culture is *not to be studied* as an object or phenomenon, nor is it about the use of different attributes to characterize groups of research participants. Rather, cultural norms and power relationships are *enacted and negotiated* through research practice or the encounter between researchers and research participants. As such, culturally responsive theories, instead of asking researchers to be culturally competent (an individual capacity, attribute, or disposition), call for them to be culturally responsive in addressing the relationality in the research process. This view is vastly different from the dominant research approaches, such as the main tenets of positivism and scientism. Rooted in Western culture and under the veil of objectivity and value neutrality, different versions of (post-) positivisms claim to be scientific, but the scientist tendency does not necessarily afford these approaches the universal validity that they claim to have when studying other cultures.

Although culturally responsive theorists endeavor to lay the foundational work for qualitative researchers to approach translation as the translation of cultures, they have, in fact, developed only limited discussions on concrete methodological interventions. The existing literature is thought-provoking in that it reveals the limitations of the current approaches, but it has yet to realize its full potential to move beyond theoretical discourses to offer practical interventions. Given culturally responsive theorists’ interest in the interrogation of cultural encounters, one may wonder if it is still necessary to delve into the specifics regarding, for example, transcription and translation, validity check methods, and styles of translation. In other words, how shall qualitative researchers grapple with the two largely distinctive approaches, namely, the translation of texts and cultures?

To envision a culturally responsive translational practice in qualitative research, we argue that it is important to maintain an intimately linked dual-focus in our discussion: On the one hand, we propose a shifting away from an exclusive focus on translation as texts as such a focus risks the dis-integration of translation from other research practices, making it harder for researchers to examine the underlying onto-epistemological congruence among them. Moreover, we notice that a root cause of the technicity in the methodological literature on translation, the tendency critiqued by culturally responsive theorists, often lies in an un-reflexive pursuit of equivalence between an original and its translated texts and is backed up by correspondence theories of meaning. Culturally responsive theories, in alignment with critical theories and post-colonial and decolonizing methodologies, offer an epistemological grounding for translation based on which qualitative researchers can perform translation as an integral part—both on the practical and onto-epistemological levels—of their research. On the other hand, we contend that the techniques and practices still matter as, however deliberately qualitative researchers develop their onto-epistemological and ethical tenets, they become impactful only if researchers enact them in their work. Culturally responsive theories facilitate the practice by raising questions about the areas of stuck-ness, illuminating the otherwise unthinkable alternatives, revealing the missed interpretations, and examining the theoretical coherence between translation and other components of research activities. More importantly, culturally responsive theories ask researchers to consider the effects of their translation, not only in the immediate context of conveying meaning among texts or utterances but also in the larger, global context of linguistic imperialism and cultural hegemony.

In what follows, we unpack our propositions in detail on three levels: the structural, the interpersonal, and the human–text interactive. As generative and iterative as qualitative research is, we have intentionally decided to avoid prescribing any checklists or procedures in our discussion. Rather, we elaborate on our methodological tenets and offer examples to illustrate them.

## Envisioning a Culturally Responsive Translational Practice

### On the Level of Social and Cultural Processes and Structure: Gatekeepers and the Routes of Translation

The discussion in this section interrogates the dominant structural power of English as the academic lingua franca. The dominant role that the English language plays in the globalized, neoliberal, and Western-centric research world has contributed to an asymmetrical translational route and resulted in reproducing social and cultural hierarchies (O’Keeffe, 2019; Vidal Claramonte, 2018). The hegemonic power of the English language is practiced through professional networks and institutionalized by academic journals and research units. As such, monolingual English communication not only conditions, filters, and gatekeeps what is considered valid qualitative research but also fuels assumptions about who are considered legitimate researchers, translators, and research participants, and what relationships are possible among them.

To further examine the relationship between language and power in the dynamic translation process, we zoom in on three stages of a typical research process: designing research, constructing/collecting data, and analyzing and reporting research findings. Languages can serve as a determining factor in the research design stage, setting up the criteria on which qualitative researchers determine an individual’s eligibility for participation in their studies. For example, after reviewing 207 articles published in the British Medical Journal (BMJ), Murray and Buller (2007) reported that only 16% of the articles mentioned language concerns. Among these 16%, more than half (nine UK studies and nine non-UK studies) used “inability to speak the language used for research” as an exclusion criterion. More recently, Ransing et al. (2023) also reported that English dominates in medical/health research across the recruitment, health information dissemination, study tool design, and reporting stages.

Furthermore, the process of designing research often requires researchers to translate English materials—from research instruments such as interview protocols to informed consent forms—into other languages, depending on the population of the research participants. At this stage, multiple institutionalized and structural factors hold researchers accountable for producing translated texts equivalent to the original texts in meaning. For instance, in several multilingual research projects that Pengfei has conducted, her institution’s ethical review board has asked her to prove the validity and fidelity of her translated research materials in different ways. In some cases, she was required to provide back translations of her translated interview protocols, and when she requested an exemption from providing back translations, she was asked to explain on what grounds she could be exempted from this mandatory procedure. In other cases, she was asked to demonstrate the linguistic expertise of her research team by providing professional references. Such practices are influenced by the common belief that translation is the search for equivalence between the original and the translated texts. Many scholars have reflected that they often need to convey the meaning of research materials (e.g., consent documents) in more participant-friendly, localized languages when in the field (e.g., Pascoe Leahy, 2022), and thus must deviate from a rigid equivalence-oriented translational practice. The default approach to translation that some institutional review board (IRB) offices have taken risks depriving a translator’s agency to adapt and localize the language used in research materials and also reinforces the assumption that researchers should primarily be accountable to rigid Western academic norms rather than to the communities being involved in the studies.

If we look at the stage at which translation is used in active data collection and analysis, it can be seen that researchers and research participants from different cultural backgrounds actively engage with each other in this contact zone, and that a research team may consist of scholars and practitioners from different cultures. Translation at this stage can be multi-directional and can shape the interactive dynamics, intersecting with other identity markers in enacting social norms and power relationships.

For example, when Wen received her doctoral training in the United States, she “studied up” (Fine, 1994) by interviewing a group of male Chinese from the elite class, primarily government officials and educational leaders, on the subject of the internalization of higher education. A “clash” of cultures took place on many levels and in many instances. During the interviews that were conducted in Chinese, the senior officials tended to “lecture” her on Chinese educational policies and practices. However, occasionally Wen would be asked to translate an English concept and explain it in Chinese. She found herself boosted with confidence and pride in those moments. At the same time, she also found herself carefully choosing wording and expressions that would not challenge the patriarchal norms embraced by this group of predominantly male interviewees. Later, when she transcribed the interviews into English, she found herself taking on a feminist perspective, questioning the interview dynamic during the transcribing process. Finally, when she ran into words whose translation she was not sure about, she would rely on her advisor—a white woman who had an intermediate proficiency in Chinese—for advice. While it is important to reflect upon Wen’s potential complicity in the cultural hegemony of English, this example demonstrates that the use of language(s) and translation is constitutive in the complex negotiation of power dynamics in research fields.

It is at the stage at which research findings are analyzed and presented in publications, presentations, and other forms of communication that the linguistic imperialism of the English language becomes fully apparent (Pennycook, 1998, 2006; Phillipson, 1992). Linguistic imperialism refers to “the dominance of English … asserted and maintained by the establishment and continuous reconstruction of structural and cultural inequalities between English and other languages” (Phillipson, 1992, p. 47). In academia, English has become a global prerequisite to teaching, research, and knowledge dissemination. Its dominant role as the default language for academic communication has rendered it a tool of imperialism that perpetuates inequalities (Pennycook, 2006). As of 2024, among the approximately 46,736 academic journals that publish papers worldwide, around 75% are published in English, amounting to about 35,000 English-language journals. Most of the 27,000 journals included in the Web of Science indexes publish in English, while more than 9000 peer-reviewed scholarly journals published in other languages (e.g., French, German, Spanish, and Chinese) are excluded from prestigious journal indexes, “perpetuating the ideology that English is the global academic lingua franca” (Curry & Lillis, 2018, para. 2). As Curry and Lillis (2018) rightly point out, scholars who do not publish in English “can’t just get their work translated,” since “it’s virtually impossible for most scholars to find translators who have a high level of academic English and know both the disciplinary content and the rhetorical conventions of academic journal articles” (Curry & Lillis, 2018, para. 4). Even for multilingual scholars, writing in a language other than their primary language(s) is a complex process that is not only time-consuming but also results in lower productivity. The invisibility of translation means that the additional labor that these researchers need to do is rarely acknowledged. Most importantly, the scholarly community is missing the opportunity to include the local knowledge and the rich meanings that can be conveyed in researchers’ primary languages. Such a loss has a ripple effect on teaching and knowledge acquisition in the broader society. As Canagarajah puts it, English is “forcing an unfamiliar pedagogical and social culture on to its learners, socio-psychologically, linguistically and politically putting them in danger of losing their first languages, cultures and identities, and contributing to the devaluation of the local knowledge and cultures” (Canagarajah, 2005, as cited in Guo & Beckett, 2007, p. 117).

In this section, we argue that translation in culturally responsive research needs to make explicit the hidden power relations between the cultures behind the source and target languages. Additionally, just being aware of the broad asymmetry and inequality is not sufficient, it is also necessary to be transparent about how a researcher’s positionality comes into being behind the choice of words, sentence structure, and logic in the translation process. Our analysis also shows that the globalized knowledge production, partly shaped by the route and gatekeepers of translation, has skewed researchers’ accountability in order to ensure that qualitative researchers’ work makes sense for the Western academic audience and that it conforms to the Western academic norms. It is thus important for culturally responsive researchers to think more deeply about how our work can serve the communities that we work with and place our accountability for these communities in the highest priority.

### On the Level of Intersubjective Relationality: Visibilizing Translators and Translation

As we explored in the previous section, translation extends beyond textual relationships; it is a practice deeply embedded in social, cultural, and political structure. In the context of cross-cultural and cross-lingual research, the role of translators deserves more examination as their work is often mentioned in passing in the final research products. Without their intermediation between researchers and research participants, cross-lingual research would be impossible.

Translation theorist Lawrence Venuti (2008) has problematized the invisibility of translators as an effect of fluent translation style:

Under the regime of fluent translating, the translator works to make his or her work “invisible,” producing the illusory effect of transparency that simultaneously masks its status as an illusion: the translated text seems “natural,” i.e., not translated. (Venuti, 2008, p. 5)

In the methodological literature on translation in social research, a translator’s role is ambiguous at best. For instance, we have not been able to locate literature that explicitly and systematically discusses the different ways that translation is incorporated into a research process (e.g., translation taking place before, during, and after data construction) and how these approaches might influence our interpretation of data differentially. Some researchers question the researcher–translator relationship (Temple & Young, 2004; Twinn, 1997), whereas others, although they emphasize the professional expertise of translators, confine their work to a research team within the scope of their translational responsibility. In health research, the role of translator/interpreter is also assumed to be neutral and, ideally, invisible. Theys et al. (2022) analyzed patients’ and interpreters’ reflections on their communicative action in patient–interpreter–doctor interactions. The findings show that interpreters expect themselves to behave as if they were invisible and to remain “impersonal” (p. 1849). However, their analysis also showed that the interpreters’ translation could influence the patient–doctor interactive dynamic, and sometimes the alternation between the original language and the translation could cause confusions on either side, effectively making the invisibility of interpreters a myth, an impossibility.

Some methodological discussions on translation presume a specific image of researchers and translators, as well as specific circumstances in which translation is needed. Some indicate that researchers encounter the challenge of translation as Western scholars, as linguistic outsiders who come to perform research *on* a community that has already been othered historically, culturally, and politically. In these cases, translators are local cultural and language brokers who connect researchers with their participants (Esposito, 2001; Squires, 2008; Temple & Young, 2004). What has been missing, especially from a critical and culturally responsive perspective, are the multiple directions in which translation can go and the potential to reverse, share, and problematize the division of labor among translators, researchers, and research participants. For instance, what does it take for a non-Western researcher, using both English and their primary language to work, to publish their work in English? How shall a space be created in our methodological framework in which a Western researcher who translates existing English research can collaborate with community-based local researchers who do not speak English? What if both researchers and participants are bilingual and transnational, and, in their communication, they use both languages interchangeably and translation takes place seamlessly?

The transparency/invisibility of the translator ironically contributes to the opaqueness of the qualitative research and reinforces a conventional route of knowledge production and circulation, that is, from the non-Western, Indigenous, and linguistically minoritized communities to English-speaking academia. Such a process may not only other the communities who are researched *on* but also those researchers who do not easily fit into the mold of white, English-speaking researchers coming from Western countries. Therefore, we argue that it is vitally important to visibilize not only translators but also the power structure that a translator is embedded in (Jijon, 2019).

Visibilizing translators and translation does not stop at understanding a translator’s position and division of labor in a research process. To continue to foster reflexivity and strive for an equitable, democratic research process, visibilizing translators should also take into account the cultural positionalities of the multiple parties involved in the research. Without that, the researchers/research team might risk missing the opportunities to interrogate the cultural differences and power relationships that are created through the negotiation of meanings in translation.

For instance, in one of the collaborative and multilingual research projects on which Pei-Jung served as a team member, the research team strove to be as transparent and deliberate as possible, but, in retrospect, when zooming in on the translation/cross-cultural, cross-lingual moments, Pei-Jung realized that the translational practices had been rendered invisible due to an adaptation—whether consciously or unconsciously—to the academic norms that prioritize research and publication in and for the English-speaking world.

The project recruited both US-based and international research participants. Given that some of the participants were in/from Asia and use Mandarin Chinese as their primary language, Pei-Jung, a bilingual Mandarin–English speaker, suggested providing Mandarin as an option for interviews. Thus, being the only international researcher and the only Mandarin Chinese speaker on the team, she assumed both a researcher’s and translator’s role throughout the research.

Among the thirty interviewees, eight were from Mandarin-speaking countries, and five of them used Mandarin in their interviews. In thematizing the main findings from the interviews conducted in Mandarin Chinese (Braun & Clarke, 2006), Pei-Jung highlighted the different yet interconnected meanings between “道德” (literal translation: morality, *dàodé*) and “倫理” (literal translation: ethics, *lúnlǐ*). She suggested including “morality” in developing one of the themes. However, the whole team eventually decided not to take up this suggestion on the grounds that “morality has a different meaning in the Western context.” The term “morality” is used by some Chinese-speaking participants to refer to a more situated ethical practice in research that is grounded in people’s everyday, culturally bounded understanding of what is right or wrong, whereas the term “ethics” is used by some Chinese-speaking participants to refer to regulatory rules. In the Western context, morality has been associated with philosophical thoughts (e.g., Hegel) and is not often linked to everyday practices. Centering the English-speaking audiences (i.e., the Western audiences), the team arrived at “personal value” as the term to use to further develop the theme.^1^

This remains an unsettling moment for Pei-Jung. At that time, she was not provided with the languages from either translational studies or culturally responsive methodology to further reflect upon her position or advocate for her view. She felt something was not right yet could not express it in her second language. Looking back, Pei-Jung finds that this moment signaled the time when the meaning differences between the original and the translated texts were “ironed out” (Temple, 2008, p. 361) in qualitative research. The imagined audience of the research was Western academics who make sense of the findings through English-speaking cultural references. This moment may reflect, as discussed in the last section, the underlying English- and Western-centric academic norms, which both the team and Pei-Jung might have unconsciously taken up at that moment. If the research team were able to conduct the analysis again, a more culturally responsive practice to “visibilize translation” might inspire Pei-Jung and her colleagues to delve deeper into the meaning differences generated through translation as well as the cultural frame behind them. Similarly, before, during, and after the translation, Pei-Jung might invite the team to create a space to explicitly document the decision-making process, to examine, for example, how the translation could be done, what the translation is for (e.g., translation style for conversation analysis and thematic analysis can differ), and how the analysis could explicate the cultural-linguistic nuances. More importantly, the whole team could work together to reflect on what it means to create a research report that makes sense to the Western audience and what they could do for those non-English-speaking participants, who represent a significant subgroup of the interviewees.

### On the Level of Human–Text Interaction: Interpretive Approaches, Untranslatability, and Styles of Translation

On the microlevel of human–text interaction, methodological decisions made by researchers and translators are often tied to their interpretative approaches. As translation moves away from a rigid pursuit of semantic equivalence, critical and culturally responsive qualitative researchers and/or translators become more intentional in approaching these decisions as conforming to or confronting English’s hegemonic structural power. As such, it is necessary to raise such questions as whether translation should be practiced or not in certain circumstances and for specific expressions, as well as into what languages and for what purpose the translation is being done (Halai, 2007). Their decisions in response to these questions can be applied to the translation of a complete piece of work, a research instrument, a large chunk of data, or a few key terms. In medical care and health research contexts, interpreters are also guided to maintain impartiality and render the original message as accurately as possible, while, at the same time, taking the cultural contexts into consideration (e.g., The National Code of Ethics for Interpreters in Health Care, 2004).

As an illustrative example, in Pei-Jung’s project described above, interpretive approaches shaped her translational practice. While translating the interview transcript, she understood the goal to be clearly and concisely communicating the participants’ main ideas in relation to the research focus. This decision was informed by the choice of the analytical approach for the project, namely, thematic analysis (Braun & Clarke, 2006). Three key features of this approach facilitated her decision-making process.

Firstly, Braun and Clarke (2006) emphasize the importance of clarifying the theoretical position of a thematic analysis, noting that the theoretical position is often left unspoken and typically deemed as a realist account by default. This emphasis informed Pei-Jung of the significance of explicating the project’s theoretical underpinning, creating space for introducing the epistemological and methodological tenets of cultural responsiveness.

Secondly, while the process of thematizing is flexible, themes must “capture something important about the data in relation to research questions” (Braun & Clarke, 2006, p. 82). This principle guided Pei-Jung’s translation decisions regarding what details to document and what to analyze succinctly. Braun and Clarke (2006) advocate for either a rich description of the entire dataset or a detailed account of a specific aspect. In this case, Pei-Jung used annotation as a tool to thoroughly describe the meaning-making process in translation and the decisions related to it.

Thirdly, thematic analysis seeks a “patterned response or meaning within the dataset” (Braun & Clarke, 2006, p. 82). Braun and Clarke state that there is no “hard and fast” rule governing the decision over what proportion of the entire data a patterned response needs to cover to be identified as a theme. Instead, they call upon a researcher’s judgment in deciding the significance of a potential theme in relation to research questions. It can be argued, then, that it is the responsibility of Pei-Jung and her colleagues to ensure that they did not overlook or misrepresent translated texts in identifying patterns, for the interest in searching for sameness among the data generated in different cultural contexts should not override the interest in being culturally sensitive and responsive in interpreting the data.

Beyond interpretive approaches, scholars who write on the general cultural phenomenon of translation have highlighted the concept of untranslatability as a form of resistance against cultural assimilation. For instance, Homi Bhabha considers the migrants’ position as one beyond the binary structure of source and target texts/places (Bhabha, 2012). The impossibility of translating migrants’ subjectivity signals “a negation of complete integration” and “a will to survival” in the society of the other (Pym, 2014, p. 140). Working in the liminal space between cultures, culturally responsive researchers have also been conducting work that can be characterized as a form of “migration”: in this case, not so much about the migration of people as about the migration of words. Acknowledging the untranslatability of some words can be one way to resist subsuming the lived experiences and lifeworld of the research participants into the interpretive framework of English-speaking academia.

Still, in Pei-Jung’s translation, a participant who is both a researcher and an IRB review board member describes the dilemma of adhering to the regulatory rules and wanting to make the researcher’s life easier. The participant used the expression “這個研究他怎麼‘交差,’” and Pei-Jung translated it as “How can he resolve this [dilemma to complete the research].” In Chinese, “交差” (*jiāo chāi*) is a term that generally refers to finishing and reporting a task. However, the meaning is context-based and multi-layered. For example, it could mean completing a task at a very minimal level and/or reporting something to someone who is considered an authority. Therefore, “交差” (*jiāo chāi*) has an implicit meaning of being accountable for something or to someone with a higher authority. What this higher authority means varies depending on the context. It could be a real authority figure such as an IRB officer, or one’s supervisor, or it could be the high order of one’s own conscience. It is hard to find an appropriate English expression to use to translate the term. Pei-Jung thus annotated: “It is a hard word to translate [for now]. We can discuss the proper term.” Although this expression is not thematized in the final report, it offers insights into future analysis of the beyond-regulation interactions between a researcher and an IRB reviewer.

A further concern within translation scholarship is the concept of fluency, a dominant translation style in contemporary English language translation wherein translators attempt to revise texts to “fit” within the dominant cultural language. By domesticating an original text in its translation (Venuti, 2008), a translator engenders a style of fluency and naturalness, and, in so doing, contributes to a subsuming of the text of the other into the receiving/dominant culture. Such an act of translation thus fuels the othering process and reinforces the existing hierarchical order among different cultures and languages. Foreignizing the translational text, on the other hand, embodies the potential to interrogate, challenge, and destabilize the existing cultural boundary, revealing its porous nature and resisting the power structure. By doing that, a translator takes up a role with greater agency in the interpretation, communication, and circulation of a foreign text. Therefore, making translation and translators visible means not only explicating and critically interrogating the very often messy and porous process of researching and translating but also diving down to the microlevel in order to understand how researchers and/or translators make decisions about each translational/interpretive task.

When translating the interview protocol from English to Chinese in her project, Pei-Jung struggled to identify an appropriate Chinese translation of the key term “ethics.” While the literal translation of “ethics” can be 倫理 (*lún lǐ*), Pei-Jung noted the nuanced differences between “ethics” and the meanings of “倫理.” For instance, “ethics” in English research contexts can encompass not only regulatory rules imposed by institutional review boards but also a broader range of moral principles in relation to research practice, whereas in Chinese, depending on specific contexts, “倫理” can be more directly associated with professional rules including research regulatory rules (e.g., those established by the IRB). Pei-Jung brought this observation up to the research team, but the team did not find an optimal way to address this cultural-linguistic nuance without altering or adding additional content to the interview questionnaire. Thus, Pei-Jung decided to include the English term “ethics” in the Chinese interview version for clarification; that is, she included the statement “In English, the term we use is ethics or ethical research” and added the description “research that is ethical to you” to better convey the meaning of “ethical research” in the Chinese translation. In doing so, Pei-Jung disrupted the fluent Chinese translation and created a sense of foreignization.

## Where Shall Qualitative Health Researchers Go From Here?

This paper began by exploring how translation is constructed and addressed in methodological literature, specifically the literature on culturally responsive methodology and the discussion on language and translation in qualitative research. What we have found are two distinctive yet intertwined approaches to grappling with the translation question: the translation of cultures and the translation of texts. The distinction between these two approaches, we argue, lies in whether researchers focus on the cultural process of translation or its final products: specifically, whether they primarily consider the macrostructural power relationships involved in translation or the microlevel interactions between humans and texts, and whether they frame translation as pressing challenges and barriers to knowledge production or as producing significant social and cultural effects that must be critically examined.

In considering the strengths and weaknesses of these two approaches, we point out that culturally responsive methodologies have empowered qualitative researchers by providing them with critical concepts and arguments with which they can interrogate the cultural and social effects of translation. Furthermore, this paper proposes that this methodological and theoretical engagement could be further substantiated through more detailed elaborations on how qualitative researchers can engage translation in their research practice. Specifically, qualitative health researchers can better cultivate reflexivity and criticality if the translation work is situated in the larger picture of global knowledge production and mobilization, the long-lived legacy of colonialism and linguistic imperialism, and the institutionalized research regulations that constantly center English-speaking academic audiences.

To make this proposal more actionable and concrete, we draw on examples from our empirical research to illustrate how qualitative researchers, especially those who are interested in practicing cultural responsiveness, can thoughtfully approach translation questions in their work on three interconnected levels: On the level of social and cultural processes and structure, qualitative researchers can examine the pathways and gatekeepers of translation in the context of knowledge production and mobilization; on the level of intersubjective relationality, it is essential to visibilize both translation and the role of translators; and on the level of human–text interaction, we suggest that qualitative researchers consider how interpretive approaches, untranslatability, and styles of translation may shape their own translation practice. It is clear that the considerations on these three levels are never separated or isolated from each other. Instead, they intersect, converge, and mutually inform one another as researchers make decisions to incorporate translation into their work by either doing the translation themselves or working with translators. Taken together, this paper outlines some important considerations that researchers, qualitative health researchers in particular, may want to take into account as they envision a culturally responsive translational practice in qualitative research, and calls for researchers to engage in this work with multilingual awareness, reflexivity, and criticality.

As we conclude this article, we hope to emphasize that this is not the end of a conversation but rather a continuation of an ongoing dialogue and an invitation to engage in further discussion. Given the rise of multilingual populations and multicultural spaces, the growing demand for decolonizing academia, and the urgent need for advocating for minority language rights in today’s world, qualitative researchers need to think with the conceptual discussions happening in culturally responsive theories and contemporary translational studies. By doing so, researchers can truly meet these social groups where they are, a responsibility that we all share.

## Note

1. While developing this current manuscript, Pei-Jung had an opportunity to revisit the choice of terminology with her research team. The team understood her concerns and incorporated the use of moral/morality in the final manuscript.
